# 36-year-old Male with Syncope

**DOI:** 10.5811/cpcem.2020.6.48419

**Published:** 2020-07-20

**Authors:** Samantha A. King, Ryan Spangler, Zachary D.W. Dezman, Laura J. Bontempo

**Affiliations:** * University of Maryland Medical Center, Department of Emergency Medicine, Baltimore, Maryland; † University of Maryland School of Medicine, Department of Emergency Medicine, Baltimore, Maryland

**Keywords:** CPC, syncope, toxicology

## Abstract

**Case Presentation:**

A 36-year-old incarcerated male presented to the emergency department (ED) after an episode concerning for syncope. The patient had nystagmus and ataxia on initial examination.

**Discussion:**

There is a broad differential diagnosis for syncope, and for patients presenting to the ED we tend to focus on cardiogenic and neurologic causes. This case takes the reader through the differential diagnosis and systemic work-up of a patient presenting to the ED with syncope.

## CASE PRESENTATION (Samantha A. King, MD)

A 36-year-old male who was currently incarcerated presented to the emergency department (ED) with a chief complaint of syncope. The patient reported that the event occurred after he stood up from dinner. There were no witnesses, but the patient believes that he hit his head. He said he had a headache since the fall, and it had not responded to the acetaminophen that he received from the prison infirmary. He denied any tongue biting or loss of bowel or bladder control. The patient stated that he had felt dizzy and lightheaded over the prior few days, and that sensation continued in the ED. He also felt numb across his shoulders and had been nauseous since the fall.

He had a past medical history of seizures and bipolar disorder. His last seizure was several years prior and was described as “whole body shaking.” The patient reported compliance with his medications, which were fluoxetine, phenytoin, ranitidine, and valproic acid. He had no prior surgical history. His family history included diabetes in his grandmother. The patient drank alcohol socially and had used marijuana and abused prescription drugs in the past but had not used any substances recently.

On physical examination, he was awake, alert, and in no acute distress. He was afebrile (36.9° Celsius) with a heart rate of 84 beats per minute, a blood pressure of 116/72 millimeters of mercury, respiration 16 breaths per minutes, and oxygen saturation of 99% while breathing room air. He was 167.6 centimeters (cm) tall and weighed 63.5 kilograms (body mass index of 22.6 kg/m^2^), and was well nourished and well developed. There was a 2 cm × 2 cm hematoma and an overlying abrasion on his left forehead. Another abrasion on his upper lip was not actively bleeding. His external ears were normal without evidence of trauma. His nose was normal. His oropharynx was clear and moist. His pupils were 3 millimeters (mm) equal, round, and reactive to light and accommodation, and eyes were without scleral icterus. His neck was supple without tracheal deviation. He had normal range of motion of his neck and he had no cervical spinous process or paraspinal muscular tenderness. His heart was regular rate and rhythm without murmurs, rubs, or gallops. He had capillary refill of less than two seconds in all extremities. His lungs were clear to auscultation bilaterally without wheezes, rhonchi, or rales. He had regular respiratory effort without accessory muscle use. His abdomen was soft with normal bowel sounds without tenderness, rebound, or guarding. There was no costovertebral tenderness. His extremities exhibited no edema, tenderness, or deformity, and had 2+ pulses throughout. He had no spinous process or paraspinal process tenderness in his thoracic or lumbar spine.

His cranial nerves (II–XII) were intact. He was found to have bilateral and direction-changing horizontal nystagmus that was provoked on lateral gaze. No vertical or torsional nystagmus was seen. He had 5/5 strength with normal muscle tone throughout his upper and lower extremities bilaterally. He had decreased sensation across his shoulders bilaterally, but the remainder of his sensation was intact. He had slow finger to nose with overshoot bilaterally. His ambulation was limited secondary to feeling unsteady. He was oriented to person, place and time, answered questions appropriately, and followed commands without difficulty.

**Image 1:**
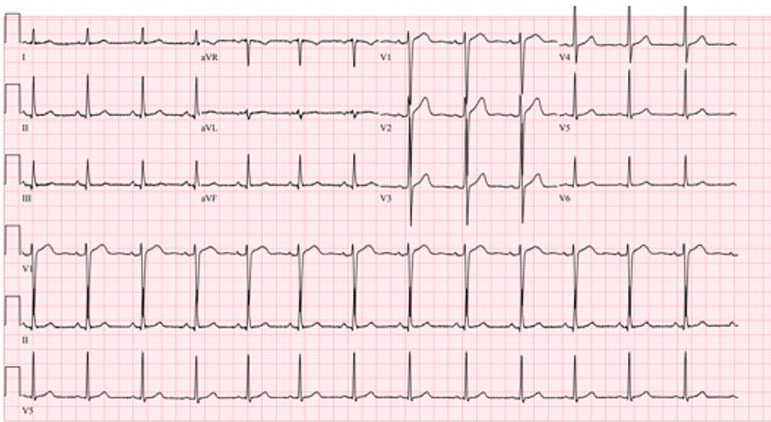
Electrocardiogram of a 36-year-old male with syncope.

**Image 2:**
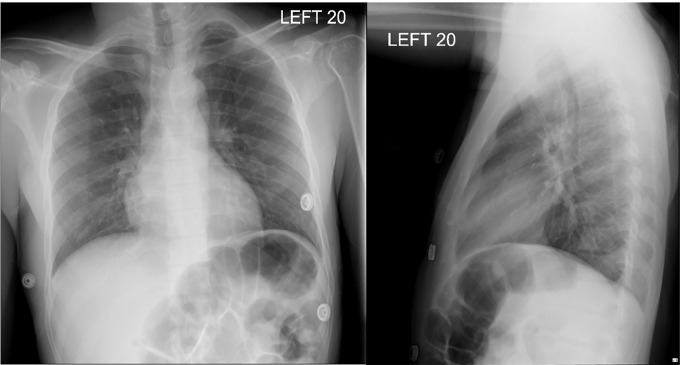
Chest radiograph posterior-anterior (left) and lateral (right) of a 36-year-old male with syncope.

Initial laboratory results are shown in Table 1. His electrocardiogram (ECG) is shown in Image 1. He had a chest radiograph (Image 2). Computed tomography (CT) of his head and neck were performed (Image 3; full study is found in Supplemental Material 1). A diagnostic test was then performed, which confirmed the diagnosis.

## CASE DISCUSSION (Ryan Spangler, MD)

The number of possible causes for this patient’s presentation was daunting. He has a range of subacute and acute symptoms, and it is challenging to determine which one is the root cause, necessitating a wide differential diagnosis. The combination of syncope and other neurologic symptoms brought to mind five categories of illness:

- Cardiovascular (syncope/dizziness)
- Primary neurologic (seizure)
- Neurovascular (stroke)
- Traumatic
- Toxicologic

The patient did not experience chest pain, trouble breathing, or other symptoms that I would attribute to atypical angina to suggest an ischemic event. The fact that he had dizziness for several days could possibly indicate persistent arrhythmia or hypotension. However, his physical exam and vital signs do not indicate signs of either of these, and his ECG confirms that he does not have an arrhythmia currently despite being symptomatic. Therefore, I eliminated a cardiovascular etiology from my differential.

Primary neurologic causes, such as seizures, would certainly be plausible in a patient with his past medical history. However, the history provided does not describe specific seizure-like activity and does not describe a notable post-ictal period. Furthermore, the patient has been compliant with his seizure medications and, from the information I have, does not have a clear reason to have a lower seizure threshold. This makes seizure an unlikely primary diagnosis.

Stroke (thrombotic, embolic, or direct vascular injury) is certainly a diagnosis that must be explored in any patient with dizziness, with particular attention being paid to the cerebellum and posterior fossa. The patient’s history included a prodrome of dizziness for several days prior to falling. It does not provide significant further information regarding the timing and triggers of the dizziness. The patient “passing out” when standing up today, supports an alternate cause being more likely than an acute stroke since ischemic strokes are unlikely to cause syncope.

The patient has nystagmus, dysmetria and ataxia, but his cranial nerve exam is normal, including full extraocular motions and equally reactive pupils. The patient also has intact strength and overall sensation, with the exception of the neck and shoulders. This exam does not support a focal cranial infarct as the etiology. Basilar artery strokes can sometimes present with several days of subacute or flow-dependent symptoms, but I would expect many more global symptoms if this were the case. While a small posterior ischemic stroke is still possible, I believe other investigation is needed.

The presentation of headache with neurologic symptoms raises concern for a subarachnoid bleed. Generally, I would expect the history of a “sudden-onset” or “thunderclap” type onset, which was not given. The CT of the head was also negative. Although lumbar puncture would be considered the gold standard test for this diagnosis, I think the likelihood of the diagnosis being an occult subarachnoid hemorrhage is unlikely based on the history provided. I was told that the patient struck his head when he passed out. This brings into question whether there is actually a traumatic injury causing some of his presenting symptoms.

His history of prodromal dizziness tends to lead me away from this; however, he complaints of, and on examination is found to have, numbness across the neck and shoulders. Injury to the cervical spine could possibly cause injury in this dermatome; however, he does not have any weakness in the upper or lower extremities, any distal sensation deficits, or tenderness on his neck exam. Overall, I think that although he does have this complaint of numbness, his overall history and exam makes it unlikely that he has a cervical spine injury.

Another traumatic etiology to consider is vertebral artery dissection since this can cause posterior neurologic symptoms such as gait instability and dysmetria. Most of his symptoms, however, are bilateral. It would be extremely unlikely for the patient to injure both vertebral arteries simultaneously. Although the patient is presenting after a fall, this likely represents a “red herring” in the case.

When considering toxicologic etiologies of the patient’s presentation, his examination is intriguing. The direction-changing horizontal nystagmus, bilateral dysmetria, and limited ambulation found on his examination are all concerning for a central neurologic injury but can also be due to other centrally acting insults, such as medication toxicity. Phenytoin and valproic acid toxicity can each present with diffuse or vague neurologic symptoms. Valproic acid toxicity typically causes tachycardia, thermal dysregulation, respiratory depression, and hypotension. Our patient has not experienced any of these effects. Phenytoin toxicity classically causes nystagmus, nausea, confusion, and ataxia. I believe this leads to the answer and can explain his bilateral neurologic symptoms.

The remaining question is this: Why would this patient have phenytoin toxicity without a recent change in dose or medication? The answer lies in his medication list. Fluoxetine and valproic acid are known to increase the systemic concentration of phenytoin due to similar cytochrome P450 metabolism, and there are case reports of both agents causing phenytoin toxicity. I believe that this interaction increased his risk of phenytoin toxicity over a longer period of time, even though there were no changes in his dosing and he was compliant. The confirmatory test will be a phenytoin level.

## CASE OUTCOME (Samantha A. King, MD)

The diagnostic test was a total phenytoin level, which confirmed phenytoin toxicity. The patient had a total phenytoin level of 27.4 micrograms/milliliter (mcg/mL). He was given intravenous (IV) fluids and ondansetron for his nausea. He was admitted to the internal medicine service, his phenytoin was held, and his phenytoin levels were trended. His phenytoin level reached a peak of 32.0 mcg/mL on hospital day (HD) 2. He thereafter had resolution of his symptoms and return of normal gait. He was ultimately discharged back to prison on HD 6. During his hospitalization, neurology was consulted. That service thought the patient’s presentation was consistent with mild phenytoin toxicity. Neurology recommended changing his valproic acid medication to alternate mood stabilizer due to concern for possible interaction. After discharge, he remained stable on his phenytoin but had other presentations to the ED for musculoskeletal injuries.

## RESIDENT DISCUSSION

Phenytoin toxicity occurs when a patient develops an excess of phenytoin in the blood related to either an acute ingestion or chronic accumulation of the drug.^1^ According to the American Association of Poison Control Centers, in 2015 there were 1606 single-agent phenytoin exposures, and of those exposures there were 33 reported “major outcomes” and two reported deaths.^2^ Phenytoin is considered one of the World Health Organization’s essential medications; and in 2016 there were a reported 2,751,980 prescriptions written for it in the United States.^3^,^4^ However, phenytoin has become less popular as other anti-epileptics have come into use and so phenytoin toxicity is expected to become less common with time. 2,4,5

Phenytoin is a voltage-gated sodium channel blocker with predominant targets in neuronal and cardiac tissue.^6^ In neuronal tissue, it particularly targets high-frequency neurons, which lends to its anti-epileptic properties. 6 It is metabolized through the cytochrome P450 system via first-order kinetics, but at higher levels it becomes metabolized through zero-order kinetics, which can be important in clearance when at toxic levels. Phenytoin is available 70% by oral ingestion and is 90% protein bound after ingestion.^6^,^7^ The high percentage of protein-bound phenytoin means that it can be greatly impacted by hypoalbuminemic states such as pregnancy and malnutrition. This fact becomes important when interpreting serum phenytoin levels. Most institutions will only have total phenytoin levels, which is typically related to the available phenytoin in the blood, but one should consider ordering a free phenytoin level if suspecting a low-protein state.^6^

Phenytoin toxicity can affect a multitude of systems including neurologic, cardiac, skin, and immunologic. The degree of neurologic toxicity occurs in relatively predictable manner in correlation to the concentration of phenytoin in the blood^6^ (Table 2). The drug levels in the patient presented here correlate with some of his physical exam findings including nystagmus and ataxia. It is also important to note that an excess of phenytoin can lead to seizures, and other anti-epileptics have also been shown to have this effect.^8^,^9^ Additionally, given that phenytoin is a sodium channel blocker, it has effects on cardiac tissue. However, this effect is rarely, if ever, seen with oral phenytoin toxicity. It is more commonly occurs with IV phenytoin toxicity, seen often with rapid infusion.^10^ These effects include QRS widening, PR lengthening, and alterations of the ST-T wave segment.^10^ Additionally, it had been thought that the propylene glycol, which is used as the diluent for phenytoin, was the only cause of these effects; however, there are case reports of cardiac effects with both phenytoin and fosphenytoin infusions.^1^ Other toxic effects of phenytoin include “purple glove syndrome,” due to vasoconstriction after IV phenytoin infusion, and hypersensitivity syndromes.^6^,^11^

Neurologic phenytoin toxicity can occur from a variety of mechanisms. A patient may have an acute toxicity secondary to either an accidental or intentional ingestion.^1^ Patients with hypoalbuminemic conditions may suffer from a chronic phenytoin toxicity.^1^,^6^ Phenytoin is metabolized through the cytochrome P450 system, allowing for many potential adverse drug interactions that can precipitate chronic phenytoin toxicity.^1^ In this case, the patient was taking valproic acid to treat his bipolar disorder. Valproic acid inhibits the P450 system, so medications like phenytoin last longer than expected in the body, which could result in a phenytoin toxicity. Lastly, phenytoin is sometimes mixed with cocaine, and there are cases in the literature of phenytoin toxicity occurring in cocaine users.^12^

Treatment of phenytoin toxicity revolves predominantly around supportive care. Fatality from phenytoin poisoning is rare, with only two deaths reported in 2015.^2^,^6^ If a patient presents acutely ill, the focus of care should be resuscitation including, if needed, airway control, cardiovascular support with fluids or vasopressors, and control of seizures using agents such as benzodiazepines and barbituates. Treatment should also be targeted at symptoms including treatment with anti-emetics and institution of fall precautions.^6^ While there is not a directed reversal or binding agent, activated charcoal has been proposed as a mechanism for possible prevention of absorption in acute ingestions.^5^ However, instead of the standard single dose, multiple doses of activated charcoal have also been proposed in a case report as a method of possible treatment.^13^ Given the protein-bound nature of phenytoin, there is current debate over the clinical effectiveness of using other mechanisms such as hemodialysis for treatment.^1^,^6^

## FINAL DIAGNOSIS

Phenytoin toxicity

## KEY TEACHING POINTS

1. Phenytoin toxicity can present with a range of symptoms and signs depending on the phenytoin level; common early symptoms include nystagmus and ataxia.
2. Phenytoin toxicity treatment focuses first on resuscitation and then supportive care.
3. Too much or too little phenytoin can cause seizures.

## Figures and Tables

**Table 1:** Initial laboratory test results of a 36-year-old male with syncope.

	Lab values	Normal values
Complete blood count		
White blood cells	4.0 K/mcL	4.5 – 11.0 K/mcL
Hemoglobin	13.9 g/dL	12.6 – 17.4 g/dL
Hematocrit	40.1%	37.0 – 50.0%
Platelets	192 K/mcL	153 – 367 K/mcL
Serum chemistries		
Sodium	138 mmoL/L	136 – 145 mmol/L
Potassium	3.9 mmoL/L	3.5 – 5.1 mmol/L
Chloride	102 mmoL/L	98 – 107 mmol/L
Bicarbonate	28 mmoL/L	21 – 30 mmoL/L
Blood urea nitrogen	10 mg/dL	9 – 20 mg/dL
Creatinine	0.7 mg/dL	0.66 – 1.25 mg/dL
Glucose	108 mg/dL	70 – 99 mg/dL
Calcium	9.1 mg/dL	8.6 – 10.2 mg/dL
Magnesium	1.6 mg/dL	1.6 – 2.6 mg/dL
Total protein	7.7 g/dL	6.3 – 8.2 g/dL
Albumin	4.3 g/dL	3.5 – 5.2 g/dL
Aspartate aminotransferase	26 u/L	17 – 59 u/L
Alanine aminotransferase	32 u/L	21 – 71 u/L
Alkaline phosphatase	84 u/L	38 – 126 u/L
Bilirubin	0.4 mg/dL	0.3 – 1.2 mg/dL

*K*, thousand; *g*, grams; *mg*, miligrams; *mmoL*, millimole; *L*, liter; *mcL*, microliter; *dL*, deciliter; *u*, units.

**Table 2:** Symptoms of phenytoin toxicity as related to total phenytoin level.^6^

Total phenytoin level	Neurologic symptoms
< 10 mg/L	Rare side effects
10 – 20 mg/L	Occasional mild horizontal nystagmus on lateral gaze (therapeutic level)
20 – 30 mg/L	Nystagmus
30 – 40 mg/L	Ataxia, slurred speech, nausea, vomiting
40 – 50 mg/L	Lethargy, confusion, hyperactivity
> 50 mg/L	Coma, seizures

*mg*, miligrams; *L*, liter.
